# Discovery of Gingipains and *Porphyromonas gingivalis* Inhibitors from Food-Derived Natural Products: A Narrative Review

**DOI:** 10.3390/foods14162869

**Published:** 2025-08-19

**Authors:** Desheng Wu, Xiaofeng Li, Guanglei Zhao, Lisha Hao, Xiaohan Liu

**Affiliations:** 1 School of Light Industry, Liming Vocational University, Tonggang West Street 298, Quanzhou 362000, China; 2 School of Food Science and Engineering, South China University of Technology, Wushan Road 381, Guangzhou 510640, China; 202220126744@mail.scut.edu.cn (L.H.); felxh6005@mail.scut.edu.cn (X.L.); 3 State Key Laboratory of Pulp and Paper Engineering, South China University of Technology, Guangzhou 510641, China; glzhao@scut.edu.cn

**Keywords:** *Porphyromonas gingivalis*, cysteine endopeptidases (gingipains, KGP), phytochemicals (natural products), polyphenols (flavonoids), structure–activity relationship

## Abstract

*Porphyromonas gingivalis* is a key periodontal pathogen whose cysteine proteases, gingipains (Rgp and KGP), are essential for nutrient acquisition and virulence. Targeting gingipains may attenuate bacterial pathogenicity and prevent related systemic diseases. This paper aimed to review advances in food-derived natural products that inhibit *P. gingivalis* or gingipains, with emphasis on mechanisms, potency, and translational potential. A literature search of several databases identified 64 studies on food-derived compounds demonstrating in vitro, in vivo, or clinical effects against *P. gingivalis* or gingipains. The results showed that tea polyphenols and dihydrochalcones (e.g., phloretin and phlorizin) inhibited gingipain activity, and a variety of food-derived natural products (especially polyphenols and polysaccharides) suppressed the growth, survival, biofilm formation, and virulence of *P. gingivalis*. Structure–activity relationships suggest galloyl moieties and dihydrochalcone scaffolds enhance gingipain inhibition. Polysaccharides and alkaloids exhibited anti-adhesion or protease inhibition, though with limited potency data. In summary, food-derived natural products represent promising gingipain inhibitors. These inhibitors have potential structure–activity relationships, indicating that food-derived natural products have considerable research prospects. Future research should prioritize structure-based discovery and structure optimization to realize their therapeutic potential.

## 1. Introduction

Based on a large-scale investigation of the Global Burden of Disease, as of 31 December 2021, the overall estimated prevalence of periodontitis in the global population was approximately 63%, with severe periodontitis estimated at 24.2%, and both trends are continuing to rise [1]. *Porphyromonas gingivalis*, a Gram-negative anaerobe, is recognized as a keystone pathogen in periodontitis and has been implicated in systemic conditions such as cardiovascular disease, Alzheimer’s disease, and rheumatoid arthritis.

Research on different strains of *P. gingivalis* has consistently shown that the virulence factors it secretes are key pathogenic factors leading to various diseases, including periodontitis. These virulence factors mainly include gingipains, fimbriae, lipopolysaccharides (LPSs), and outer membrane vesicles (OMVs), which play important roles in the survival, invasion, host immune regulation, and inflammation induction of *P. gingivalis*. Among these, gingipains are considered the most critical virulence factors, and many complications caused by *P. gingivalis* are closely related to their action [2,3]. Studies have shown that knocking out the gingipain genes in *P. gingivalis* significantly reduces the virulence of the strain [4]. Notably, *P. gingivalis* requires the secretion of gingipains to obtain iron and protoporphyrin IX for its metabolism, presenting an important breakthrough point for research on treatment strategies against this bacterium [5].

The survival and pathogenicity of *P. gingivalis* are closely related to its virulence factors. Its cysteine proteases, gingipains—arginine-specific (RgpA and RgpB) and lysine-specific (KGP)—are essential for nutrient acquisition, host tissue destruction, and immune modulation [6,7,8]. By degrading host structural proteins, cytokines, and complement factors, gingipains orchestrate both local and systemic inflammation, making them attractive therapeutic targets [8,9].

Food-derived natural products, characterized by structural diversity and favorable safety profiles, have emerged as promising gingipain inhibitors. In addition to directly inhibiting bacteria and biofilm formation, compounds such as epigallocatechin gallate, phloretin, and phlorizin can inhibit KGP and/or Rgp activity. Compounds such as proanthocyanidins and resveratrol can effectively reduce inflammation caused by *P. gingivalis*. However, most findings are based on in vitro assays, with limited mechanistic elucidation, scarce structure–activity relationship analyses, and few in vivo validations. Furthermore, challenges such as poor bioavailability, instability under physiological conditions, and a lack of clinical evidence hinder translation to therapeutic use. To date, no comprehensive review has focused specifically on food-derived gingipain inhibitors or their chemical diversity, mechanisms, and translational prospects.

This review aims to systematically summarize advances in food-derived natural products that inhibit *P. gingivalis* or gingipains, with an emphasis on chemical classes, potency, and mechanisms of action. By integrating recent evidence, including structure–activity relationship insights, this study critically evaluates their therapeutic potential and discusses strategies to overcome pharmacokinetic and delivery challenges. We provide a foundation for the future research and development of gingipain-targeted interventions derived from dietary sources.

## 2. Literature Search Strategy

A comprehensive literature search was conducted to identify relevant studies investigating the inhibitory effects of natural products on gingipains, key virulence factors of *Porphyromonas gingivalis*. The electronic databases PubMed, Web of Science, and Google Scholar were systematically searched for articles published between January 2000 and December 2024.

The search strategy combined MeSH terms and commonly used keywords, namely, “*Porphyromonas gingivalis*,” “gingipain,” “KGP,” and “periodontitis,” together with terms related to natural compounds, namely, “inhibitor”, “natural products,” “phytochemical,” “polyphenol,” “flavonoid,” “polysaccharide,” “oligosaccharide,” “alkaloid,” “terpenoid,” “quinone,” “saponin,” and “lignan.” Boolean operators (AND/OR) were used to refine the search.

Studies were included if they met the following criteria: (1) original research articles including in vitro or animal experimental studies; (2) a clearly defined chemical structure and natural source for the tested compounds or extracts; (3) evaluations of inhibitory effects on gingipain activity or related virulence mechanisms.

The exclusion criteria comprised the following: (1) non-English language articles; (2) review articles without experimental validation; (3) studies not involving specific natural products; (4) articles whose findings have been superseded by more recent and comprehensive publications.

**Figure 1:**
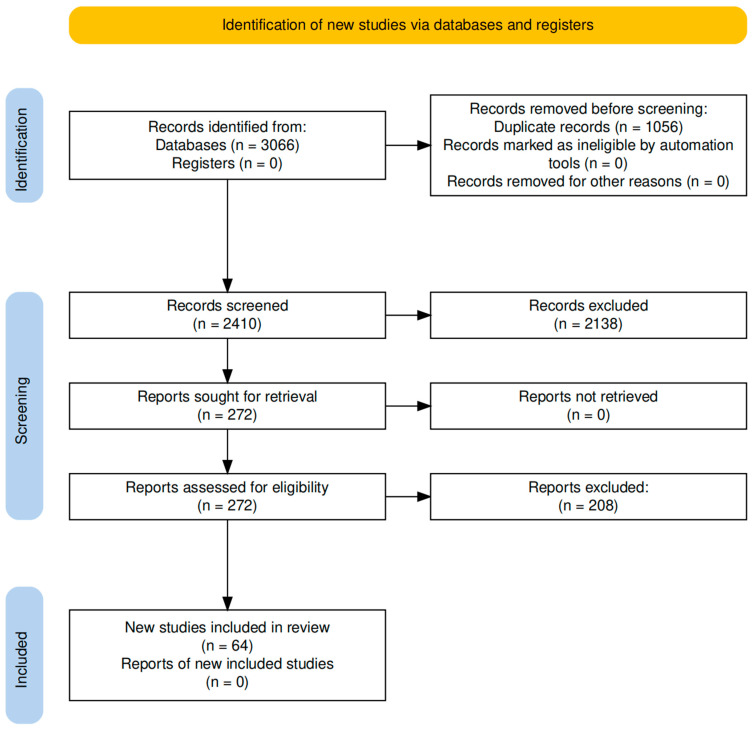
PRISMA 2020 flow diagram illustrating the study selection process for this review. The diagram was created using the PRISMA2020 flow diagram R package v1.1.3 [11].

The study selection process is illustrated in Figure 1, according to the PRISMA guidelines [10].

## 3. Overview of *Porphyromonas gingivalis*

### 3.1. Biological Characteristics and Systemic Implications of P. gingivalis

*P. gingivalis* requires heme and vitamin K for growth, allowing it to thrive and rapidly proliferate in the presence of blood [12]. Research findings suggest that *P. gingivalis* has immune evasion abilities, enabling it to interfere with inflammatory signaling, disrupt the complement system, control apoptosis, and undermine host defense mechanisms, thereby creating conditions for other pathogenic factors to act [13].

*P. gingivalis* selectively manipulates host responses and inhibits bactericidal mechanisms without suppressing inflammation. The tissue degradation products released during inflammation, such as peptides and heme, become important nutrient sources for *P. gingivalis* and some oral bacteria [14]. This increase in pro-inflammatory factors triggers bone loss and ultimately results in periodontal tissue destruction and alveolar bone resorption [15].

**Figure 2:**
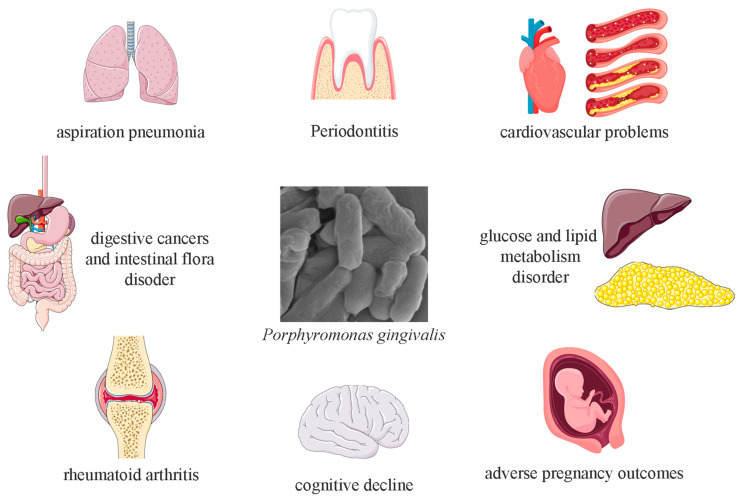
Overview of health problems related to *Porphyromonas gingivalis*. Parts of the figure were created using open-source images from Servier Medical Art and Vecteezy, and all are licensed.

*P. gingivalis* possesses strong penetrative capabilities, with its virulence factors increasing the permeability of cellular barriers, allowing it to even penetrate the blood–brain barrier. *P. gingivalis* can survive not only in the oral cavity but also in other organs, contributing to the onset and progression of systemic diseases such as cardiovascular disease, cognitive decline, adverse pregnancy outcomes, type 2 diabetes, and rheumatoid arthritis (Figure 2) [16,17,18,19]. Additionally, eating behaviors can facilitate *P. gingivalis*’s entry into the digestive tract along with food, potentially inducing gastrointestinal diseases and altering the composition of the gut microbiota [20].

**Figure 3:**
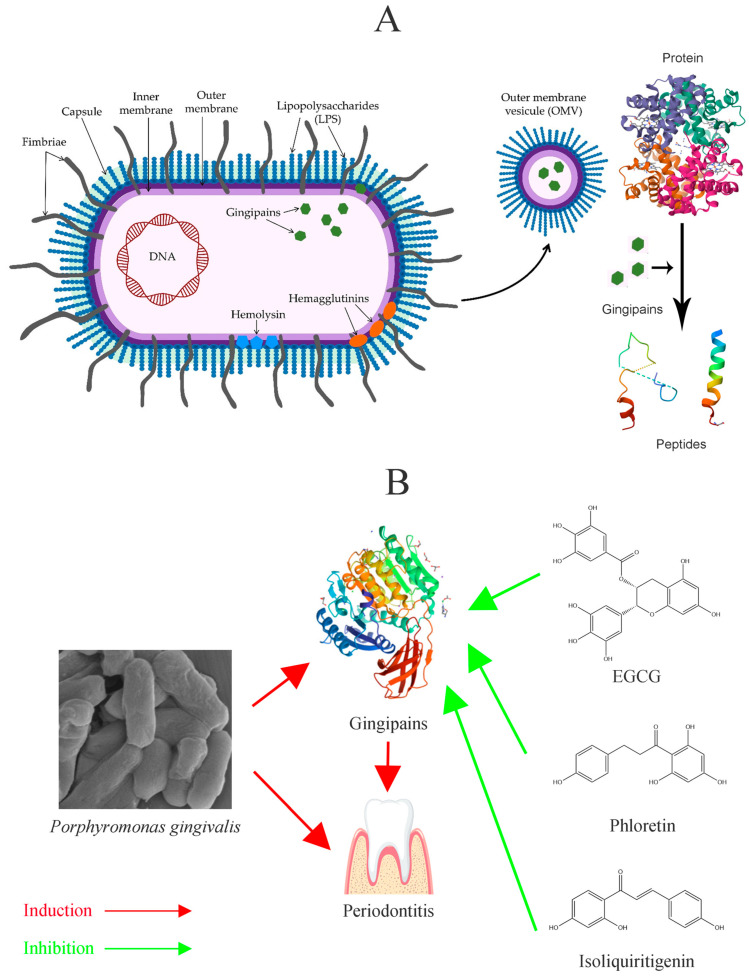
(**A**) shows the structure of *Porphyromonas gingivalis* and its virulence factors. Parts of the figure used pictures from a publication by Aleksijević [2]. (**B**) shows the relationship between *Porphyromonas gingivalis*, gingipain, and periodontal disease; some natural products can improve periodontal disease by inhibiting gingipain.

Gingipains are central to the colonization, disruption of host defenses, tissue destruction, and nutrient acquisition of *P. gingivalis* (Figure 3A) [2,12]. The survival and pathogenicity of *P. gingivalis* are largely dependent on its virulence factors; a lack of gingipains significantly hampers its growth and reduces its pathogenic potential (Figure 3B). Beyond gingipains, LPSs primarily induce inflammatory responses and bolster the bacterium’s immune evasion capabilities [21,22].

### 3.2. The Roles of Gingipains in the Survival and Pathogenicity of Porphyromonas gingivalis

#### 3.2.1. Overview of Gingipains

Gingipains are multifunctional enzymes involved in the colonization, nutrient acquisition, and manipulation of host immune systems by *P. gingivalis*, making them indispensable tools for this bacterium. They are considered its most critical virulence factors. Gene knockout, antigen immunization, and targeted inhibition of gingipains have all been shown to effectively reduce the pathogenicity of *P. gingivalis* [7].

Gingipains are the primary proteolytic enzymes secreted by *P. gingivalis*. These cysteine proteases account for over 85% of its total proteolytic activity [23]. Gingipains include arginine-specific gingipains (Rgp), divided into RgpA and RgpB, and lysine-specific gingipain (KGP). The genes encoding these enzymes are conserved across multiple strains of *P. gingivalis*. These three enzymes can either function as membrane-bound proteins or be secreted into the external environment in a soluble form. RgpA and RgpB share essentially the same protease and immunoglobulin-like domains, but RgpB is secreted as a monomer, whereas RgpA forms a stable hemagglutinin–adhesin (HA) domain, similar to KGP [24]. Inhibiting any of these functional domains would have a significant impact on the survival and pathogenicity of *P. gingivalis*.

#### 3.2.2. Role of Gingipains in the Survival of *Porphyromonas gingivalis*

*P. gingivalis* needs to adhere to the host surface to complete colonization, and it also needs to co-aggregate with other oral bacteria to colonize the biofilm. This adhesion requires the joint action of fimbriae and gingipains. The HA domains on RgpA and Kgp are required for the adhesion function of gingipains [25]. When these domains are lost, or when gingipain is lacking, the co-aggregation ability of *P. gingivalis* is significantly reduced.

In addition to adhesion, gingipains are crucial for *P. gingivalis* in obtaining nutrients. *P. gingivalis* is a porphyrin auxotroph, and heme is an ideal source for both iron and porphyrin. As such, the bacterium relies on gingipains to capture sufficient heme [6]. Studies have shown that RgpA and KGP can bind hemoglobin with high affinity, and once hemoglobin is anchored near the protease domains, it is gradually degraded and releases heme [6,26]. This hemolytic property is directly linked to the activity of gingipains. During this process, Rgp and KGP work in tandem: Rgp facilitates the conversion of hemoglobin into a form more easily degraded by KGP, and KGP then efficiently completes the degradation. The absolutely conserved HA-2 domain on gingipains captures the degraded heme and directs the secreted gingipains to capture more hemoglobin [27]. This function is lost in mutant strains in which the HA domain is knocked out.

Experimental evidence shows that when gingipains are inhibited, *P. gingivalis* cannot grow in media supplemented with transferrin. However, when the supplement is switched to heme, the growth of *P. gingivalis* is restored [28]. This characteristic provides a potential direction for the development of *P. gingivalis* inhibitors.

#### 3.2.3. Role of Gingipains in the Pathogenicity of *Porphyromonas gingivalis*

Gingipains also play an important role in *P. gingivalis*’s resistance to host immune system responses. The complement system is one of the main players in the human body’s resistance to oral bacterial invasion, and *P. gingivalis* relies on gingipains to resist complement-mediated lysis [29]. The HA domain of RgpA can bind to the human complement inhibitor C4b-binding protein to form protective capabilities [30]. At the same time, all three gingipain types can degrade complements C3, C4, and C5, rendering their defense systems ineffective. In this process, RGP performs significantly better than KGP [9]. In addition to antagonizing the complement system, gingipains can also protect *P. gingivalis* from immune cell attacks and even destroy cell surface receptors and weaken the immune system’s communication network [9].

In addition to ensuring *P. gingivalis* viability, gingipains also play a critical role in *P. gingivalis* aggressiveness. In a manner independent of proteolytic activity, RgpA and KGP manipulate cytokine networks to help *P. gingivalis* evade host defenses and alter the local environment [8]. This gives *P. gingivalis* the ability to control inflammatory responses, which is beneficial to both offense and defense. The host’s coagulation cascade, protease-activated receptor cascade, and kinin pathway are inhibited by gingipains, paralyzing the host’s defense system, assisting *P. gingivalis* in breaking through the vascular barrier to spread, and reversing the protective effect of the inflammatory response to the host’s own attacks [31]. Although gingipains have proteolytic activity, they may not directly degrade periodontal tissue. In fact, they inactivate the host’s protease inhibitors, disrupt the balance between host proteases and endogenous protease inhibitors, and allow the host’s proteases to attack periodontal tissue regardless of friend or foe, ultimately leading to alveolar bone resorption and periodontal ligament destruction [7]. Inducing host cell apoptosis with gingipain not only enhances its destructive effect on periodontal tissue but also enhances the invasion and spread ability of *P. gingivalis* [32].

Research has found that gingipains can enhance the bacterium’s ability to penetrate the blood–brain barrier by degrading intercellular junction proteins [33,34]. Peripheral infections play a significant role in cognitive decline and the progression of Alzheimer’s disease, and gingipains have been detected in substantial numbers in the brains of AD patients [3].

### 3.3. The Structure of Gingipains

#### 3.3.1. Structure of RGP

Gingipain-R is encoded by two genes, *rgpA* and *rgpB*, and divided into two types of enzymes, RgpA and RgpB [35]. These enzymes are multidomain proteins: RgpA consists of a catalytic domain, plus four additional HA domains between the immunoglobulin superfamily domain (IgSF) and the C-terminal domain to form a non-covalent complex, HRgpA, and RgpB lacks the HA domain, only appears in the catalytic domain [36]. The difference between RgpB and RgpA is not only related to the HA domain, although the catalytic domain of RgpB is similar to that of RpgA; four amino acid substitutions around the active site of RgpA allow it to degrade protein substrates more efficiently [37]. The loss of RgpB has specific effects on RgpA maturation, and the loss of RGP can also cause abnormal KGP processing. Therefore, although alveolar bone loss is significantly induced only when *P. gingivalis* expresses RgpA, the role of RgpB cannot be ignored [37]. The structure of RgpB is the main direction of RGP structure research, and most studies related to the HA domain tend to analyze it in combination with KGP.

**Figure 4:**
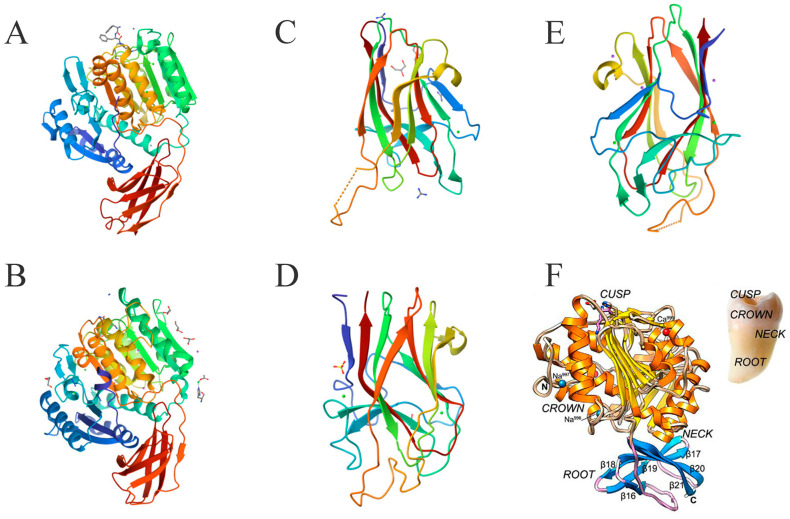
Crystal structure of gingipains. RgpB ((**A**), PDB ID: 1CVR) consists of four domains. KGP consists of a catalytic domain ((**B**), PDB ID: 4RBM), a K1 Cleaved Adhesin Domain ((**C**), PDB ID: 4ITC), a K2 Cleaved Adhesin Domain ((**D**), PDB ID: 3KM5), and a K3 Cleaved Adhesin Domain ((**E**), PDB ID: 3M1H). (**F**) is a comparison of KGP with tooth structure. Parts of the figure use images from a publication by de Diego [39].

The structural shape of RgpB is similar to a single-rooted tooth. The crown formed by the 351 N-terminal residues is the catalytic domain, and the root formed by 84 residues is similar to the IgSF domain (Figure 4A). The catalytic domain of RgpB consists of two subdomains, A and B, and the active center of RgpB is located on the “chewing surface” of the “tooth crown” [38]. The center is exposed Cys244, surrounded by a pocket formed by His211-Glu214, Ala243-Val245, and Glu152-Asp163. The active site residues are inferred to be His211 and Cys244, and Glu152 also has a significant influence. The catalytic domain of RgpB is highly similar to that of caspase, and it is speculated that the catalytic mechanism of RgpB may be similar as well. The S1 pocket of RgpB can preferentially accommodate Arg side chains, and structural differences provide it with a higher optimal pH, necessitating an active anaerobic environment [38]. The function of the IgSF domain for RgpB is less obvious because this domain is mainly responsible for anchoring the HA domain, so it would be more significant for RgpA [36].

#### 3.3.2. Structure of KGP

KGP is encoded by the *kgp* gene and is a multi-domain protein consisting of a signal peptide, N-terminal pro-domain (NPD); a catalytic domain, IgSF; three to five HA domains; and a C-terminal domain. Abnormal function in the *kgp* gene does not affect the expression, processing, or activity of RGP [40]. KGP and RGP are sensitive to lysine and arginine, respectively, making them cleave proteins differently. The folding structure of the Kgp catalytic domain is similar to that of Rgp, especially in the NPD, but they share less than 23% sequence identity (Figure 4A,B) [41]. By contrast, the NPD sequence identity of RgpA and RgpB reaches 75% [42]. KGP is similar to RgpA in that it forms a non-covalent complex with the HA domain during post-translational events, shaping its unique function [43].

The catalytic domain of KGP consists of CD and IgSF. Interestingly, its structure is very similar to that of a tooth, with CD as the crown, IgSF as the root, and the active site located in the tooth cusp (Figure 4F) [39]. Structural analysis of the catalytic domain of KGP shows that the catalytic pocket of its active site is actually a catalytic triplet formed by Cys477-His444-Asp388, rather than a cysteine–histidine duplex [39]. A structure–activity mechanism analysis of the targeted inhibitory effect of KYT-36 inhibitors on KGP has also proved this [4]. The C-terminal domain of KGP has an extended solvent channel that passes through the inner core of the protein, which not only enhances the plasticity and flexibility of the enzyme but also increases its energy cost due to instability [39]. This structural difference weakens the proteolytic ability of KGP, making its protein catalytic activity lower than that of RgpB [44]. However, the actual role of KGP is far greater than that of RgpB, because the complex with the HA domain is the key for KGP to cover the functions of heme acquisition, delivery, and binding to the extracellular matrix in support of *P. gingivalis*.

#### 3.3.3. Structure of Haemagglutinin/Adhesin Domains

Structural differences in the modular composition and organization of the HA domain provide KGP with several structural variants, giving *P. gingivalis* strains different functional properties. By contrast, the conservation of RgpA in different *P. gingivalis* strains is as high as 99% [45]. The HA domain contains three types, K1, K2, and K3, which can also form complexes with each other (Figure 4C–E). Since the *P. gingivalis* W83 strain can express all three HA domains, it is an excellent sample for studying HA domains and also one of the most virulent [46].

The cleaved HA domains K1, K2, and K3 share 30% sequence identity, with the sequence identity of K1 and K3 being 71%. Their folds share significant similarities, especially in the core fold, and the short-loop end of their β-sandwich is highly conserved in sequence and conformation terms (Figure 4B–D) [46]. The loop conformations at the long-loop end are significantly different, but this seems to provide the opportunity for their interaction [47]. Arg1109 of K1 in L9, Arg1280 of K2 in L8, and Arg1557 of K3 in L10 are their arginine anchor sites, and their spatial structures are very similar [46]. Analysis of their surface electrostatic potential shows that the electron density of the Thr1098–Ser1104 region on loop L9 of K1 is weak or missing [46]. The electron density of the three fragments in loop L8 of K2, Lys1276, Arg1280, and Lys1291, is weak or missing, and the lysis or truncation of this region severely abrogates the hemolytic capacity [48]. K3 has weak or missing electron density in the Leu1544–Pro1553 region of L10, in which the density of Leu1544–Lys1547 is weak, and the electron density of Thr1551–Ala1552 and Ala1546–Pro1553 is missing [47].

K1, K2, and K3 can all selectively bind hemoglobin with high affinity and induce hemolysis in vitro, but heat treatment renders this hemolytic ability ineffective [46,47,48]. The lack of a catalytic domain indicates that hemolysis is not related to proteolytic activity. The anion transport inhibitor SITS can reduce the detectable hemolysis threshold of K2 by one order of magnitude and potentially the hemolysis mechanism of HA domains [48]. Crystal structure and hemolytic performance tests show that there may be functionally relevant structural differences between K1, K2, and K3.

## 4. Current Treatment Options for *Porphyromonas gingivalis* Related Problems

Currently, the treatment of periodontal diseases associated with *P. gingivalis* infections primarily involves physical debridement, irrigation therapy, and antibiotics [49]. Chlorhexidine is a commonly used antibacterial mouthwash in clinical practice, and nonsteroidal anti-inflammatory drugs (NSAIDs) may be used to relieve inflammation. Metronidazole, doxycycline, minocycline, amoxicillin, and clindamycin are the most commonly used antibiotics [50]. However, due to the long treatment cycles and high recurrence rates of periodontal diseases, antibiotics can only serve as a short-term treatment option. Prolonged antibiotic use can disrupt beneficial microbiota in the oral cavity or body and the development of antibiotic resistance. Moreover, when biofilms form subgingivally, the effectiveness of antibiotic therapy is significantly reduced compared with planktonic cells [51]. Antibiotic resistance in *P. gingivalis* has been observed in multiple regions and populations, making the development of new drugs that are effective, safe, and less prone to causing resistance an important measure to address these issues [52].

Antibiotics and chemical drugs such as chlorhexidine have sufficient clinical data to support their use, but they are not routinely used in every case. Developing specific inhibitors targeting *P. gingivalis* or discovering more effective broad-spectrum antimicrobial agents are two important approaches to addressing *P. gingivalis*-related issues. Studies have shown that targeting gingipains can help reduce the risk of diseases associated with *P. gingivalis* [3,53].

Given the crucial role of virulence factors such as gingipains in the pathogenicity of *P. gingivalis*, targeted inhibition of these virulence factors is also a viable option. However, there are no reports on the targeting inhibition of gingipain activity by antibiotics. The complex structure of gingipains also poses challenges to the development of their targeted inhibitors. This has led to slow progress in research on *P. gingivalis* and gingipain inhibitor discovery over the past two decades.

Studies have developed molecular-targeted inhibitors to solve the problem of *Porphyromonas gingivalis* and its virulence factors, achieving effective results in animal experiments, such as with KTY inhibitors and COR inhibitors, but there have been no reports of successful clinical applications [3,4,54].

Natural products, widely distributed in nature with a diverse range of types and biological activities, represent a treasure trove for the development of new functional food factors and novel drugs [55]. Many natural products exhibit broad-spectrum antibacterial properties, including some with inhibitory effects against *P. gingivalis*. Given this, there is great potential in discovering targeted inhibitors of gingipains. The safety of food-derived natural products also provides a strong guarantee for their long-term application.

**Figure 5:**
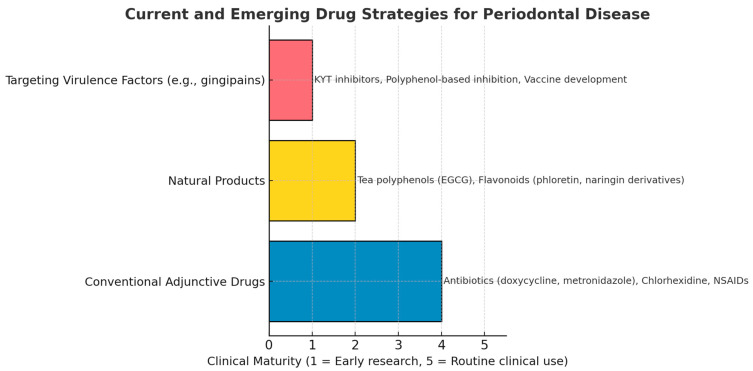
Clinical maturity of pharmacological strategies for periodontal disease. The treatment options listed in each category are examples only.

However, to date, most natural products and inhibitors targeting virulence factors remain in animal experiments or early clinical trials (Figure 5).

## 5. Discovery of Gingipain Targeted Inhibitors and Potential Structure-Activity Relationships

Given the critical importance of gingipains to the survival and pathogenicity of *P. gingivalis*, the development of targeted inhibitors is essential. RgpA, RgpB, and KGP are the three most important gingipains, with RgpA and KGP exhibiting significantly higher toxicity, likely due to their HA domains. Overall, Rgp tends to play a supportive role, while KGP is the primary contributor to virulence and is crucial for the hemoglobin cleavage that allows *P. gingivalis* to acquire heme. Targeting the structure of gingipains to inhibit their function is an effective way to reduce the pathogenicity of *P. gingivalis* and even decrease its population, with research on KGP being the most critical direction. The structure–activity relationships found in studies related to gingipain inhibition can also help to discover targeted inhibitors.

One study examined the effects of catechins and their derivatives on Rgp and KGP activities. It found that catechin derivatives, including EGCG, (−)-epicatechin gallate (ECG), (−)-gallocatechin gallate (GCG), and (−)-catechin gallate (CG), significantly inhibit Rgp activity; however, only GCG and CG significantly inhibit KGP activity, and their performance is worse in inhibiting Rgp [56]. In this experiment, only tea catechin derivatives containing the galloyl moiety inhibited Rgp and KGP activities, indicating that this mechanism may be associated with the presence of a galloyl moiety linked to 3-OH. This provides an important reference for studying the structure–activity relationship of catechins in inhibiting gingipain, but further evidence is still needed to advance research in this area.

Another study tested the anti-inflammatory effects of 18 dihydrochalcones and structurally related compounds on *P. gingivalis* LPS-induced periodontitis. The structure–activity analysis results indicated the importance of electronegative atoms on the A and B-linker chains for the anti-inflammatory effect of flavonoids, suggesting that, in addition to interactions with NF-κB transcription factors, these molecules may have direct molecular interactions with virulence enzymes, providing a reference for the discovery and structural improvement of leading compounds [57].

**Figure 6:**
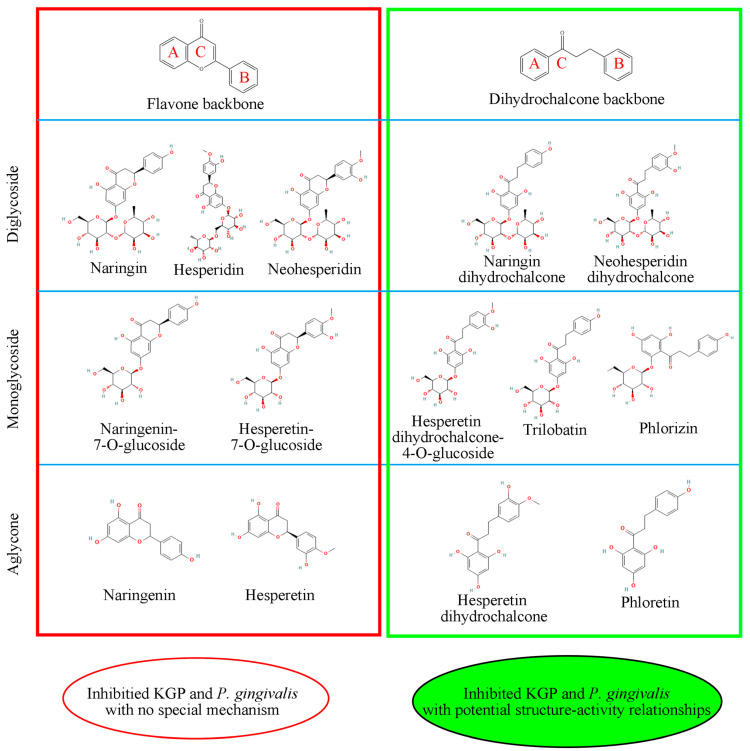
A series of flavones and dihydrochalcones reported to inhibit KGP with potential structure–activity relationships [28].

**Figure 7:**
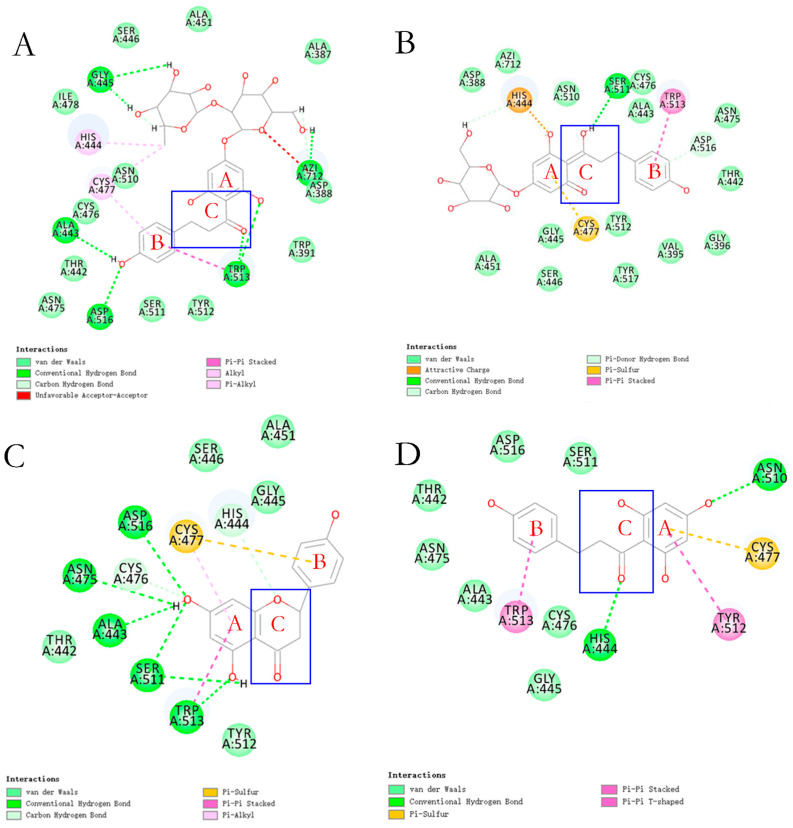
Molecular docking revealed the possible binding mode of dihydrochalcone compounds with KGP [28]. (**A**) is naringin dihydrochalcone, (**B**) is phlorizin, (**C**) is naringenin, and (**D**) is phloretin. The key structure of the compounds are marked in the figure.

In recent studies based on the reported structure of KGP crystals, 14 flavonoids were identified with the ability to inhibit gingipain activity through molecular docking and virtual screening, demonstrating a significant structure–activity relationship (Figure 6 and Figure 7) [28]. Among these, flavonoids with a dihydrochalcone structure demonstrated stronger inhibitory effects and interfered with *P. gingivalis*’s utilization of hemoglobin [28]. However, when the dihydrochalcone structure transformed into a flavonoid structure, as the C ring closed, this interference effect disappeared, and their antibacterial effect was significantly reduced. Among these flavonoids, phloretin and phlorizin performed best. However, the effect of naringin dihydrochalcone was poor, which may be due to steric hindrance caused by the diglycoside group.

## 6. Potential Antibacterial Agents Against *Porphyromonas gingivalis* from Food-Derived Natural Products

Some natural products that are abundant in common foods could inhibit *P. gingivalis*, presenting the possibility of substituting medication with dietary intake (Table 1 and Table 2). However, most studies on the antibacterial effects of natural products on *P. gingivalis* have not discussed the role of gingipains. Problems related to *P. gingivalis* may be solved by inhibiting virulence factors such as gingipains and lipopolysaccharide, and it is worth further analyzing the specific mechanism. A comprehensive analysis of related studies will facilitate the identification of lead compounds within natural products that could resolve issues associated with *P. gingivalis* and gingipains, potentially discovering structure–activity relationships.

### 6.1. Polyphenols

**Figure 8:**
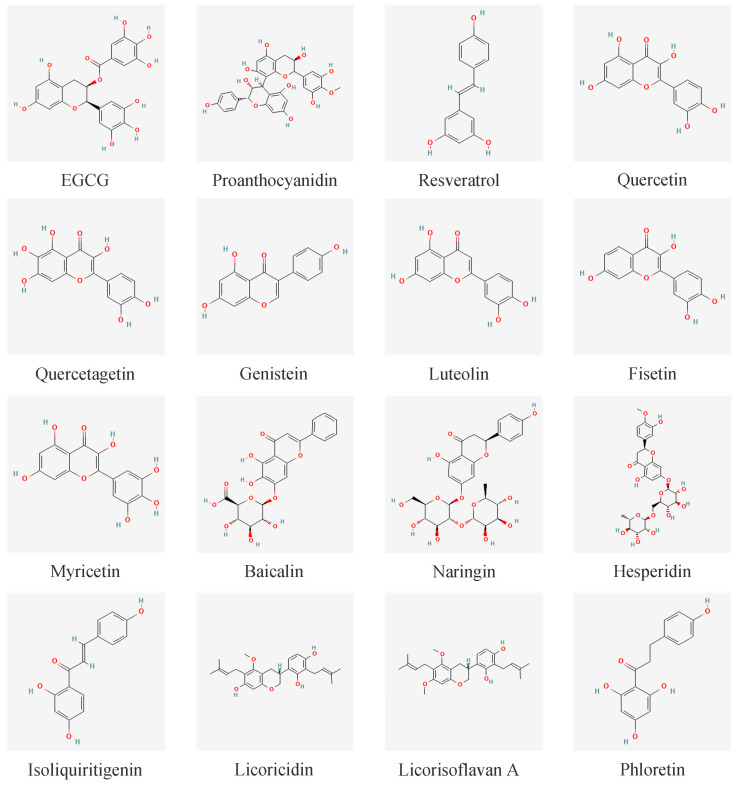
Structure of polyphenols that with the ability to address *P. gingivalis*-related problems.

In research on *P. gingivalis*-related natural products, polyphenols such as flavonoids occupy a considerable proportion and have obvious effects. Among them, tea polyphenols, proanthocyanidins, resveratrol, and several flavonoids have significant effects. The structures of polyphenols with inhibitory effects are summarized in Figure 8.

#### 6.1.1. Tea Polyphenols

Epidemiological surveys have revealed a modest inverse association between green tea intake and the incidence of periodontal disease [58]. Daily green tea intake was significantly correlated with multiple periodontal health indicators; the more frequently the subjects drank green tea, the better their periodontal condition. Antibacterial experiments on clinically isolated strains have also verified that *P. gingivalis* is sensitive to green tea extract, and 12.5 mg/mL green tea extract can have a significant inhibitory effect on the growth of *P. gingivalis* [59]. The main component of green tea polyphenols is epigallocatechin-3-gallate (EGCG). Confirmed by several studies, EGCG has a direct antibacterial effect, damaging the cell membrane and biofilm of *P. gingivalis*. However, when the biofilm is constructed, the inhibitory effect of EGCG may be greatly reduced [60].

Administering EGCG to mice with periodontitis can effectively inhibit the activation of the NF-κB signaling pathway through *P. gingivalis* virulence factors, downregulate the levels of various inflammatory mediators, and alleviate alveolar bone loss caused by inflammation [61]. In one experiment, EGCG administration for 40 days reduced elevated levels of IL-1β, IL-6, IL-9, and IL-12p70 induced by *P. gingivalis*; it also inhibited RANKL-induced activation of the JNK/c-Jun and NF-κB pathways, IL-1β-induced bone resorption, and osteoclast formation but had no significant regulatory effect on IL-17, which can induce RANKL activation. However, whether the regulatory effects were associated with any virulence factors was not verified.

Studies on osteoclasts and gingival fibroblasts have shown that, by inhibiting IL-6 and blocking the activation of the MAPK/ERK signaling pathway, EGCG can reduce the expression and activity of matrix metalloproteinase-1 (MMP-1), MMP-2, and MMP-9 induced by *P. gingivalis* LPS, thereby preventing alveolar bone resorption [62]. A study on ligature-induced periodontitis SD rats also demonstrated that, by reducing the level of IL-6, systemic administration of EGCG for more than 2 weeks can reduce osteoclast activity and inhibit collagen destruction, providing it with a potential therapeutic effect on damaged periodontal tissue [63]. In-depth research on the anti-infection mechanism showed that EGCG can induce gingival epithelial cells to secrete the antimicrobial peptide human β-defensin and protect them from degradation caused by gingipains, enhancing β-defensin’s establishment of innate immune defense against gingival cells and its role in promoting wound healing [64]. The use of specific protein kinase inhibitors has demonstrated that the mechanism by which EGCG induces β-defensin secretion involves activating the ERK/MAPK pathway.

The anti-inflammatory effect can also reduce the systemic inflammation problems caused by *P. gingivalis* virulence factors and reduce various health risks, such as atherosclerosis [65]. This effect mainly involves inhibiting EGCG regarding increased levels of inflammatory factors and peroxidation damage caused by *P. gingivalis*’s virulence factors [65]. This means that the antioxidant function of dietary polyphenols may help alleviate oxidative damage induced by *P. gingivalis* virulence factors, thereby contributing to the relief of symptoms.

Some studies have used total catechins or green tea extracts for *P. gingivalis* research instead of pure EGCG. Besides reducing IL-1β by inhibiting NF-κB, MAPK, and the TLR pathway and activating NLRP3 and AIM2 inflammasomes to reveal anti-inflammatory effects, the total catechin in green tea also synergizes with traditional antibiotics such as metronidazole to enhance its ability to inhibit *P. gingivalis*, interfering with AI-2 signaling [66,67]. Moreover, the expression of several important genes involved in host colonization (*fimA*, *hagA*, and *hagB*), tissue destruction (*rgpA* and *kgp*), and heme acquisition (*hem*) has shown significant catechin dose-dependent inhibition [66].

Given the research results for green tea, the inhibitory effects of white tea, oolong tea, and black tea extracts on *P. gingivalis* have also been studied. No significant differences have been observed, although the catechin content and proportion of catechin derivatives in these four teas are different [68]. They can all interfere with the growth and toxicity of *P. gingivalis* and exert anti-inflammatory effects. All four tea extracts strongly inhibit the collagenase activity of *P. gingivalis*, which may interfere with its acquisition of protein nutrition.

Research results for black tea show that theaflavins also attenuate *P. gingivalis* toxicity, inhibit the activity of KGP and Rgp, inhibit inflammatory signaling pathway activation, promote β-defensin secretion, and inhibit MMP-1 and MMP-2 expression [69,70,71].

The systemic bioavailability of small-molecule catechins is high, that of galloyl catechins is low, and the oligomers and polymers in black tea are extremely low or nonexistent, which provides guidance for the utilization and structural modification of tea polyphenols [72].

#### 6.1.2. Berry Polyphenols

Most of the research related to *P. gingivalis* and proanthocyanidins comes from studies of cranberry polyphenols. In addition to green tea, berry extract has also been found to have mitigating effects on *P. gingivalis*-related issues. In a study using cranberry, wild blueberry, and strawberry complex extracts, the growth, adhesion, and toxicity of *P. gingivalis* were inhibited [73]. Although the berry-mixed extract had poor antibacterial performance, with an MIC of more than 2000 mg/L, the hemolytic activity of *P. gingivalis* was completely inhibited at a concentration of 31.25 μg/mL, and the activities of collagenase, Rgp, and KGP were reduced by 35.8%, 84.2%, and 67.6%, respectively. Analysis of the components of the mixed extract showed that phenolic acids, flavonoids (flavonols, anthocyanins, and flavan-3-ols), and proanthocyanidins accounted for 10.71%, 19.76%, and 5.29%, respectively. This provides an important molecular structure reference for discovering targeted inhibitors of gingipains.

Several studies have tested the effects of polyphenolic components in cranberry extract on *P. gingivalis*-related issues. They found that these polyphenols can interfere with the formation of biofilms and bacterial coaggregation, reduce adhesion capability, inhibit the activation of inflammatory factors such as IL-6 caused by *P. gingivalis* virulence factors, suppress the secretion of MMP-2 and MMP-9, and exhibit inhibitory effects on gingipains [74,75,76]. Sánchez et al. found that the amount of *Porphyromonas gingivalis* was reduced by 39.3% after exposure to 20 mg/mL of cranberry extract for 60 s, while the use of 0.2 mg/mL cranberry extract for 6 h could reduce colonization by 97.2% [74]. However, an analysis of the extract components showed that its main components were benzoic acid, p-coumaric acid, protocatechuic acid, and flavan-3-ol A-type trimers; the anthocyanin content was not high, and the experiment did not test the efficacy of virulence factors. Neto et al. conducted an experiment to classify the components of cranberry polyphenols and concluded that the antibacterial and anti-adhesion effects of cranberry on *P. gingivalis* mainly came from the combined effects of its high-molecular-weight nondialyzable material (NDM) fraction and other polyphenols, rather than from proanthocyanidins [75]. Yamanaka et al. found that the total cranberry polyphenol components, other than NDM, inhibited the activity of Rgp and KGP at 1 mg/L; furthermore, the inhibition rate could reach 87–91% at a concentration of 100 mg/L, but they believed that the activity mainly came from the EGCG component rather than proanthocyanidins [77]. Tanabe et al. demonstrated that the effect of cranberry extract on *P. gingivalis* LPS-induced osteoclast formation and bone resorption originated from proanthocyanidins (especially A-type cranberry proanthocyanidins), which could inhibit RANKL-dependent osteoclast differentiation by 95% and inhibit the secretion of MMP-2 and MMP-9 [76]. Notably, the anti-inflammatory effects of proanthocyanidins have been widely confirmed in past studies. Research on *Pelargonium sidoides* DC extracts has verified the inhibitory effect of proanthocyanidins on *P. gingivalis* [78,79]. These benefits may come mainly from proanthocyanidins in the berry, but they lack the ability to inhibit proteases. However, considering that the method used to extract the NDM component increases proanthocyanidins up to 125 times compared with cranberry juice, the inhibitory effects of proanthocyanidins on gingipain cannot be ruled out, and more evidence is needed [80]. Currently, there is a lack of structure–activity relationship research on the inhibition of *P. gingivalis* using berry polyphenols, and we look forward to future progress in this field.

In vitro and in vivo studies have shown that berry polyphenols have low bioavailability and are particularly susceptible to conversion into other metabolites caused by intestinal microbiota [81]. The high variability in data between subjects suggests that their bioavailability depends on the intestinal microbiota, the food matrix, and possible interactions with other components of the diet.

#### 6.1.3. Resveratrol

Resveratrol has also shown promising results for *P. gingivalis*-related problems. Resveratrol is an antitoxin naturally produced by plants and present in various foods, and it is an important mechanism of bacterial resistance. Many studies have confirmed the anti-inflammatory and antioxidant effects of resveratrol and its good safety profile [82].

Resveratrol has a direct antibacterial effect on *P. gingivalis*, destroying biofilms and reducing the adhesion ability of bacteria [83]. According to Ben Lagha et al., at a concentration of 250 µg/mL, resveratrol can completely inhibit *P. gingivalis*, inhibiting 51.59% of biofilm activity and 59.91% of adhesion ability. At the same time, 40 μg/mL of resveratrol treatment for 2 h can inhibit 48.84% of the collagen degradation caused by *P. gingivalis*. O’Connor et al. found that resveratrol inhibited the anaerobic bacteria *P. gingivalis* and *Actinobacillus actinomycetemcomitans* but not other aerobic microorganisms [84]. This means that resveratrol’s antibacterial effect may be a result of its antioxidant properties, which interfere with the relevant metabolic pathways of anaerobic bacteria that shape the anaerobic environment [84]. At the same time, pterostilbene, an analog of resveratrol, can increase intracellular reactive oxygen species (ROS), and this conclusion may provide new insights into its antibacterial mechanism and structure–activity relationship [82]. The specificity of resveratrol against anaerobic bacteria makes it less likely to cause drug resistance in oral bacteria.

The anti-inflammatory effect is the main way resveratrol relieves health problems caused by *P. gingivalis*. The increased levels of inflammatory factors IL-1β, IL-6, IL-8, IL-12, TNF-α, and NO induced by *P. gingivalis* LPS can be inhibited after applying resveratrol; the reduction ranges from 40% to 80.2%, and the mRNA expressions of IL-6, IL-8, IL-1β, IL-12, and TNF-α are highly inhibited, reflecting its powerful anti-inflammatory ability [85]. At the same time, the inflammatory pathway signal transduction triggered by *P. gingivalis* virulence factors, such as LPS, can also be significantly inhibited and partially blocked by resveratrol. The expression of TLR4 decreases, and the phosphorylation of downstream factors NF-κB p65, p38MAPK, and STAT3 is inhibited [86]. Furthermore, resveratrol and its derivative activate the downstream expression of ERK1/2, AMPK, and SIRT1, inhibiting the activation of the NF-κB pathway and the expression of inflammatory factors [87]. This effect can be achieved by treating the target cells with low concentrations (1–25 μM) of resveratrol for 30 min. Interestingly, although SIRT1 activation is one of the mechanisms by which resveratrol exerts its therapeutic effect, knocking out SIRT1 does not counteract its anti-inflammatory effect [88]. The inhibition of the NF-κB signaling pathway with resveratrol may be the most important mechanism, but it is independent of SIRT1. Activating NF-κB induces osteoclast differentiation, and resveratrol contributes to inhibiting alveolar bone loss caused by osteoclast differentiation [89]. In one study, resveratrol preferentially inhibited pathological bone resorption but did not impair physiological bone metabolism, as the expression of related genes did not differ between the blank and treatment groups, whereas the expression of *Il1b*, *Ptgs2*, and *Tnfsf11*, which are related to osteoclast differentiation and activation, was significantly inhibited.

This powerful anti-inflammatory effect helps alleviate symptoms other than periodontal disease caused by *P. gingivalis*, including rheumatoid arthritis and enhanced vascular permeability caused by VEGF overexpression [90]. The effect of resveratrol on rheumatoid arthritis may be due to the fact that resveratrol can restore the level of IL-4, which is decreased by antagonizing the elevated IL-17 levels induced by *P. gingivalis* [90]. This is not only beneficial for protecting periodontal tissues but can also alleviate joint inflammation and bone destruction.

In addition to anti-inflammation effects, the expression of virulence-factor-related genes *fimA*, *kgp*, and *rgpA* has also been inhibited by resveratrol [91]. This not only helps reduce the colonization of *P. gingivalis* but also indirectly reduces the problems caused by gingipain toxicity. However, further evidence is still needed to prove whether resveratrol has the ability to inhibit the activity of Rgp and KGP directly.

In vitro and in vivo studies have accumulated extensive data on the bioavailability of resveratrol. Resveratrol’s poor water solubility and rapid metabolism contribute to its low bioavailability [92]. Human studies have shown that the amount of free resveratrol entering the bloodstream increases linearly with increasing doses, while the *T_max_* value remains constant [93]. This suggests that a specific dose of resveratrol is appropriate, taking into account its bioavailability and the low risk of potential side effects.

#### 6.1.4. Flavonoids

Flavonoids are polyphenolic compounds derived from plants. Past studies have reported their anti-inflammatory, antioxidant, and antibacterial applications. There are also studies on the use of a variety of flavonoids on *P. gingivalis* [94]. These effects cover many factors and include various issues related to *P. gingivalis*, including antibacterial qualities, biofilm inhibition, inhibiting the activation of inflammatory pathways, reducing inflammatory factors, repairing oxidative damage, and regulating the differentiation of osteoblasts/osteoclasts to protect alveolar bone.

Studies have reported the effects of quercetin, genistein, luteolin, and quercetagetin on *P. gingivalis* LPS-induced inflammation, showing that they can inhibit the expression of the MAPK, ERK1/2, p38, and JNK pathways, and reduce multiple inflammatory factors, especially cyclooxygenase-2 (COX-2), with quercetagetin performing the best [95]. Research on quercetin has shown that quercetin not only directly inhibits *P. gingivalis* but also reverses the inhibition of osteoblast differentiation caused by LPS by regulating MAPK signaling [96]. Luteolin inhibits the activation of the MAPK, AKT, and NF-κB signaling pathways and NOS expression, abolishes LPS’s effects on NF-κB translocation, and reduces alveolar bone resorption [97,98]. Similarly to luteolin, fisetin and myricetin can also inhibit the activation of these signaling pathways and block LPS-induced COX-2 expression [99,100]. According to Grenier et al., 100 μg/mL of myricetin can decrease the expression of *fimA*, *hagA*, and *hagB* by 81%, 93%, and 64%, respectively, and the expression of *rgpA*, *rgpB*, and *kgp* by 68%, 49%, and 96%, respectively. This could highly inhibit collagen degradation [100].

In addition to regulating MAPK, NF-κB, and TLR signaling, baicalin also regulates macrophage polarization to clear LPS-induced inflammation by inhibiting the RhoA/ROCK signaling pathway; it also has a good protective effect on osteoblasts and alveolar bone [101,102]. Hesperidin, widely found in citrus fruits, can also inhibit *P. gingivalis* and reduce inflammation [103]. Maquera-Huacho et al. reported that hesperidin dose-dependently inhibits *P. gingivalis*-induced ROS production and the secretion of IL-1β, TNF-α, IL-8, MMP-2, and MMP-9; it also attenuates the activation of NF-κB in *P. gingivalis*-stimulated macrophages, thereby protecting the gingival epithelial barrier [104].

Isoliquiritigenin, licoricidin, and licorisoflavan A isolated from licorice can also reduce the toxicity of *P. gingivalis*, and licochalcone A can synergize with A-type cranberry proanthocyanidins to reduce the secretion of pro-inflammatory mediators [103,105,106]. Notably, isoliquiritigenin and liquiritigenin are very similar in structure, but the opening on the C ring of isoliquiritigenin makes its anti-inflammatory properties superior to those of liquiritigenin. Applying 5 μg/mL of isoliquiritigenin can reduce TNF-α by 87% and block LPS-induced CCL5, IL-6, and IL-1β secretion, while the same concentration of liquiritigenin can only inhibit LPS-induced TNF-α, IL-1β, IL-6, IL-8, and CCL5 by 17%, 34%, 23%, 11%, and 24%, respectively. This indicates a potential structure–activity relationship. Another study investigated the antibacterial effects of prunin laurate and its analogs on *P. gingivalis* [107]. The lauroyl chain moiety of prunin laurate is essential for growth inhibition and could be associated with the bacterial membrane, and physical damage to the cell membrane is not the cause of growth inhibition.

Recent studies have demonstrated the significant potential of phlorizin and phloretin in inhibiting *P. gingivalis* and gingipains [28,108]. In addition to targeted inhibition of gingipains, transcriptomics-based studies have revealed that phlorizin, phloretin, and naringenin can all inhibit the transposition function of *P. gingivalis* [108]. This provides important guidance for dealing with the drug resistance of *P. gingivalis*.

In summary, flavonoids have great potential in reducing *P. gingivalis* toxicity and inhibiting inflammatory reactions, and they have an obvious structure–activity relationship, which deserves further exploration.

Flavonoids readily interact with other food components, complicating the study of their absorption mechanisms. Furthermore, flavonoids are utilized by intestinal microbiota and then metabolize into other products, reducing their blood concentrations [109]. In vitro and in vivo studies have shown that flavonoid aglycones have higher bioavailability than glycosides, which are often metabolized to aglycones before exerting their effects. However, the natural concentration of aglycones in plants is low, making dietary intake difficult.

### 6.2. Polysaccharides and Oligosaccharides

There are few studies on the inhibitory effects of polysaccharides and oligosaccharides on *P. gingivalis*. The findings are mostly focused on reducing adhesion ability, and only a few studies report direct antibacterial effects and enzyme inhibitory abilities. Polysaccharides extracted from *Panax ginseng* exert strong anti-adhesive activity against oral pathogens in a dose-dependent manner at concentrations of 0.1–2.0 mg/mL; partial hydrolysis of the polysaccharide into oligosaccharides retains anti-adhesive activity, but enzymatic hydrolysis results in a complete loss [110]. This anti-adhesive activity has also been observed in *P. gingivalis*-related studies on *Glycyrrhiza glabra* polysaccharides and *Rhododendron ferrugineum* leaf polysaccharides, reducing the adhesion of *P. gingivalis* by 60% and 75%, respectively [111,112]. In addition, *Rhododendron ferrugineum* polysaccharide has a time- and dose-dependent inhibitory effect on the activity of Rgp but had no effect on KGP, and an inhibitory effect on hemagglutinin was also observed. Exopolysaccharides (EPSs) from *Lactobacillus plantarum* EIR/IF-1 postbiotics inhibit the auto- and co-aggregation of *P. gingivalis*, exhibiting antibiofilm activity, which may be due to reduced bacterial hydrophobicity, thereby inhibiting bacterial aggregation [113]. In addition to having an IC_50_ of 5 mg/mL against *P. gingivalis*, Jujube polysaccharides (JPs) not only reduce the adhesion ability but also the invasion ability and cytotoxicity of *P. gingivalis*. This can lead to significant changes in the composition of the oral microbiome, potentially reshaping the oral microbiota [114]. However, the complex structure of polysaccharides makes it difficult to explore structure–activity relationships.

Interestingly, drug-free and non-crosslinked chitosan scaffolds can induce the formation of *P. gingivalis* cell clusters and exhibit direct antibacterial activity. As chitosan can be used to make antibacterial films and hydrocolloids and enhance the antibacterial properties of materials, the composite material formed by cross-linking chitosan and small-molecule inhibitors may represent a new development direction for *Porphyromonas gingivalis* antibacterial agents [115,116,117].

These polysaccharides have only been reported in in vitro experimental data, and no data related to bioavailability and pharmacokinetics are available.

### 6.3. Others

Berberine extracted from *Coptidis rhizoma* has a direct antibacterial effect on *P. gingivalis* but can also reverse the downregulation of osteogenesis-related gene expression in bone mesenchymal stem cells (BMSCs) caused by *P. gingivalis* [118,119]. It can also inhibit collagenase activity. The expression of osteogenic-related genes such as OSX, COLI, ALP, OCN, and OPN significantly increases after berberine treatment, showing a combination of antibacterial effects and promotion of osteogenic differentiation. Wnt signaling-specific inhibitor DKK-1 can block the effect of berberine, indicating that its mechanism of action is involved in regulating the Wnt signaling pathway. Berberine is already a registered drug, and the results of related pharmacokinetic studies have been made public. However, due to its poor fat solubility and poor gastrointestinal absorption, its bioavailability is low, and increasing the dosage may lead to toxicity risks.

*Corydalis saxicola* bunting total alkaloids (CSBTA) can block the pyroptosis of macrophages caused by *P. gingivalis* LPS [120]. The alkali-transformed saponin extracted from quinoa shell has an antibacterial effect on *P. gingivalis*, and reducing the polarity of the saponin can improve its antibacterial performance. In addition, the leakage of cell components suggests that its mechanism of action may be the destruction of bacterial cell membranes [121].

Some studies have explored the effects of natural extracts on *P. gingivalis*-related issues but not the specific components responsible for their effects. For example, aqueous extract from the leaves of *Rhododendron ferrugineum* has demonstrated the ability to inhibit Rgp, and *Copaifera reticulata* oleoresin has demonstrated antibacterial activity against various oral pathogens, including *P. gingivalis* [112,122]. The acetone–water extract of *Limonium brasiliense* (Lb) roots can inhibit gingipain activity and adhesion; the reduced bacterial adhesion is due to a strong interaction between proanthocyanidins and gingipains [123]. Natural products extracted from *Boswellia trees* (*B. serrata*) have also shown antimicrobial activity against *P. gingivalis* growth and biofilm formation [124]. The peel extract of *Garcinia mangostana* L. not only has an antibacterial effect but also has a significant inhibitory effect on biofilm formation. The author of one study speculates that this function may come from its xanthone derivatives, flavonoids, and saponins [125].

**Table 1:** Parts of polyphenols that are shown to be active against *P. gingivalis* and its virulence factors.

Nature Products	Type	Food Source	Mechanism	Virulence Factors	Model	References
EGCG	Polyphenol	Tea	Antibacterial, anti-inflammation, reduces virulence, alleviates alveolar bone loss, reduces invasion ability, enhances immune system defense, inhibits gingipain expression	LPS, KGP, Rgp, *kgp*, *rgp*, *fimA*	A, O	[60,61,62,63,64,65,66,126]
Catechin	Polyphenol	Tea	Anti-inflammation	/	A	[67]
Theaflavins	Polyphenol	Tea	Antibacterial, anti-inflammation, reduces virulence, alleviates alveolar bone loss, enhances immune system defense	KGP/Rgp	O	[69,70,71]
Proanthocyanidin	Polyphenol	Berry	Anti-inflammation, reduces virulence, alleviates alveolar bone loss	LPS	O	[74,75,76,77,78,127]
Resveratrol	Polyphenol	Grapes, peanuts	Anti-inflammation, inhibits virulence factor expression	LPS	A, O	[85,86,87,88,91,128]
Quercetin/ Quercetagetin	Flavonoids	Potato leaves, pea leaves	Antibacterial, regulates MAPK pathway, protect alveolar bone	LPS	A, O	[95,96,129,130,131]
Luteolin	Flavonoids	Peanut shells	Inhibits MAPK, AKT, and NF-κB signaling pathways; reduces alveolar bone resorption	LPS	A, O	[97,98]
Fisetin/ Myricetin	Flavonoids	Berry, apple	Inhibits MAPK, AKT, and NF-κB signaling pathways; blocks COX-2 expression	LPS, *kgp*, *rgp*, *fimA*	O	[98,99,100]
Baicalin	Flavonoids	*Scutellaria baicalensis*	Inhibits MAPK, NF-κB, TLR, and RhoA/ROCK signaling pathways; protects osteoblasts and alveolar bone	LPS	A, O	[101,102,132]
Hesperidin	Flavonoids	Citrus peel	Antibacterial, anti-inflammation	LPS	O	[104]
Isoliquiritigenin/ Licoricidin/ Licorisoflavan A	Flavonoids	Licorice	Inhibits NF-κB p65 and activator protein-1	LPS	O	[103,105,106]
Phloretin/ Phlorizin	Flavonoids	Apple	Antibacterial, inhibits KGP activities	KGP	O	[28]
Naringenin	Flavonoids	Citrus peel	Antibacterial, inhibits KGP activities	KGP	O	[28]

Note: In the model column, “A” indicates animal studies, and “O” in vitro studies.

**Table 2:** Parts of natural products (excluding polyphenols) that are shown to be active against *P. gingivalis* and its virulence factors.

Nature Products	Type	Food Source	Mechanism	Virulence Factors	Model	References
PG-F2/PG-HMW	Polysaccharide	*Panax ginseng*	Anti-adhesion	/	O	[110]
*Glycyrrhiza glabra* polysaccharides	Polysaccharide	*Glycyrrhiza glabra*	Anti-adhesion	/	O	[111]
RF-RPS	Polysaccharide	*Rhododendron ferrugineum*	Anti-adhesion	KGP, Rgp, *kgp*, *rgp*, *fimA*	O	[112]
EIR/IF-1 EPSs	Polysaccharides	*Lactobacillus plantarum* EIR/IF-1	Anti-adhesion	/	O	[113]
JP	Polysaccharide	Jujube	Anti-adhesion, reduces invasion ability and cytotoxicity, reshapes oral microbiota	LPS	O	[114]
CS	Polysaccharide	Shrimp shells, crab shells	Adsorbs bacterial cells	/	O	[115]
Berberine	Alkaloid	*Coptidis rhizoma*	Antibacterial, restores osteogenesis-related gene expression	/	O	[118]
CSBTA	Alkaloid	*Corydalis saxicola* Bunting	Blocks macrophage pyroptosis	LPS	O	[120]
QS/ATS	Saponin	Quinoa	Destroys bacterial cell membrane	/	O	[121]

Note: In the model column, “O” indicates in vitro studies.

## 7. Conclusions

This study is an overview of *P. gingivalis* and gingipains and their hazards, summarizing relevant research on natural products for treating related problems. The structural characteristics of gingipain indicate that targeting specific domains to inhibit its function is an effective way to reduce the pathogenicity and abundance of *P. gingivalis*.

Antibiotic treatment represented by metronidazole is currently the main treatment for *P. gingivalis* related problems. Substitution with food-derived natural products can effectively complement the shortcomings of antibiotic treatment. A survey of relevant studies shows that polyphenols, represented by tea polyphenols, cranberry polyphenols, resveratrol, and various flavonoids, have good therapeutic effects, especially in inhibiting inflammatory reactions and alleviating alveolar bone loss. Epigallocatechin gallate, phloretin, and phlorizin can inhibit KGP and/or Rgp activity, and structure–activity relationships suggest that galloyl moieties and dihydrochalcone scaffolds enhance gingipain inhibition. Clear structure–activity relationships will make research on gingipains more realistic.

To address the common problem of low bioavailability in polyphenol compounds, esterification modification may be considered to improve their bioavailability; nanoparticle encapsulation may also be used to enhance delivery performance and reduce the impact of intestinal microbiota.

Notably, few relevant studies of natural products have explored the structure–activity relationship between their structures and gingipain inhibition. These products represent promising gingipain inhibitors, with potential structure–activity relationships indicating that food-derived natural products have considerable research prospects. Understanding of structure–activity relationships will inspire structure-based virtual screening and greatly improve the efficiency of researchers in discovering potential inhibitors.

However, due to a lack of evidence from clinical practice, the summary of natural products in this article mainly focuses on in vitro tests and animal studies. The complex in vivo environment and differences between species will have a great impact on the practical application of these natural products. More original research is needed to prove the feasibility of developing inhibitors derived from natural products. Future research should prioritize structure-based discovery and structure optimization to realize their therapeutic potential, focusing on the in vivo application of these natural products.
